# Switching first-line targeted therapy after not reaching low disease activity within 6 months is superior to conservative approach: a propensity score-matched analysis from the ATTRA registry

**DOI:** 10.1186/s13075-020-02393-8

**Published:** 2021-01-06

**Authors:** Lucie Nekvindová, Jiří Vencovský, Karel Pavelka, Pavel Horák, Zlatuše Křístková, Jakub Závada

**Affiliations:** 1 Institute of Biostatistics and Analyses, Ltd., Brno, Czech Republic; 2 Institute of Rheumatology, Na Slupi 4, 128 00 Prague, Czech Republic; 3 Department of Rheumatology, First Faculty of Medicine, Prague, Czech Republic; 4 Department of Internal Medicine III - Nephrology, Rheumatology, Endocrinology, Faculty of Medicine and Dentistry, Palacký University, Olomouc, Czech Republic; 5 University Hospital, Olomouc, Czech Republic

**Keywords:** Registry, Treat-to-target, Rheumatoid arthritis, Propensity score

## Abstract

**Background:**

Treat-to-target (T2T) is a widely accepted strategy for patients with rheumatoid arthritis (RA). It recommends attaining a goal of at least low disease activity (LDA) within 6 months; otherwise, the current therapy should be modified. We aimed to investigate whether switching a first-line targeted therapy (TT) in patients not reaching LDA within 6 months leads to a higher probability of meeting LDA at the 12-month visit in daily clinical practice using data from Czech registry ATTRA.

**Methods:**

We included patients with RA starting the first-line TT from 1 January 2012 to 31 January 2017 with at least 1-year follow-up. We created four mutually exclusive cohorts based on (1) switching to another TT within the first year and (2) reaching a treatment target (DAS28-ESR ≤ 3.2) at the 6-month visit. The primary outcome was the comparison of odds for reaching remission (REM) or LDA at the 12-month visit between patients switching and not switching TT after not reaching treatment target at 6 months. Before using logistic regression to estimate the odds ratio, we employed the propensity score to match patients at the 6-month visit.

**Results:**

A total of 1275 patients were eligible for the analysis. Sixty-two patients switched within the first 5 months of the treatment before evaluating treatment response at the 6-month visit (C1); 598 patients reached the treatment target within 6 months of therapy (C2); 124 patients did not reach treatment response at 6-month visit and switched to another therapy (C3), and 491 patients continued with the same treatment despite not reaching LDA at the 6-month visit (C4). We matched 75 patients from cohort C3 and 75 patients from C4 using the propensity score. Patients following the T2T principle (C3) showed 2.8 (95% CI 1.4–5.8; *p* = 0.005) times increased likelihood of achieving REM/LDA at the 12-month visit compared to patients not following the T2T strategy (C4).

**Conclusions:**

In daily clinical practice, the application of the T2T strategy is underused. Switching TT after not reaching REM/LDA within the first 6 months leads to a higher probability of achieving REM/LDA in RA patients at the 12-month visit.

## Background

Currently, patients with rheumatoid arthritis (RA) have multiple drug options with different mechanisms of actions to address the heterogeneous nature of the disease. Patients may require multiple successive therapies throughout their lives. In 2010, the European League Against Rheumatism (EULAR) developed its first recommendations for the management of rheumatoid arthritis with synthetic and biological disease-modifying anti-rheumatic drugs (DMARDs) [1]. There were several updates throughout the years with the last update so far at the end of 2019 [2]. Treating toward a target of remission (REM) or at least a low disease activity (LDA) has become the standard of care for patients. Achievement of the treatment target often requires switching between drugs. According to EULAR recommendations, therapy with conventional synthetic (cs) DMARDs should be started as soon as the diagnosis of RA is made, and methotrexate (MTX) should be the first choice. If the treatment target is not reached with the first csDMARDs, and poor prognostic factors are present (i.e. presence of rheumatoid factor/anti-citrullinated protein antibodies, high disease activity early, joint damage, failure of two or more csDMARDs), a biological (b) DMARD or targeted synthetic (ts) DMARD should be added. If there is no improvement within 3 months after the start of treatment or if patients have not reached the treatment target with bDMARD/tsDMARD by 6 months, therapy should be adjusted, and treatment with another bDMARD or tsDMARD should be considered [2].

The approach currently recommended for RA treatment involves titrating medication dosages until pre-specified disease activity targets (either REM or LDA) have been met and maintaining these targets over time. Such so-called treat-to-target strategies (T2T) have proven to be more effective and to generate better outcomes than usual care [3, 4]. The efficacy of the T2T approach has been evaluated in many randomised controlled clinical trials [5–11]. Even though the T2T strategy has been widely applied in daily clinical practice nowadays, studies from daily clinical practice concerning the advantage of following T2T over usual care are still required. Several studies evaluating the efficacy of T2T in real clinical practice have been already done [4, 12–17], but more evidence through real-life data is needed to support the implementation of T2T.

The primary aim of this study was to assess whether following a T2T strategy after not reaching treatment target (REM/LDA) within the first 6 months leads to a higher probability of meeting the treatment target at the 12-month visit in daily clinical practice. We also described four groups of patients based on different courses of their treatment with the first bDMARD/tsDMARD.

## Methods

### Study setting and data source

The ATTRA registry, established in 2002, is a prospective, national, observational cohort study. Its main purpose is to evaluate the safety and effectiveness of bDMARDs (and lately also tsDMARDs) in patients with chronic inflammatory rheumatic diseases. Patients with RA (and ankylosing spondylitis, psoriatic arthritis and juvenile idiopathic arthritis) starting bDMARDs or tsDMARDs are recruited from fifty practice sites (private or academic), which captures more than 95% of patients with RA treated with bDMARDs/tsDMARDs in the Czech Republic (CZ). Targeted therapy (TT) has been reimbursed for patients with RA if DAS28 > 5.1 despite therapy with csDMARDs until 2019. Since 2019, the cut-off for DAS28 was lowered to 3.2 in CZ. Initial TT should include either TNF inhibitors (TNFis) or tsDMARDs. At the time of this analysis, the ATTRA database included information on 5050 patients with RA.

At the start of therapy, baseline data are collected including demographics (gender, age at diagnosis, age at the start of first-line treatment, height, weight, smoking status, presence of comorbidities), disease characteristics (disease duration, presence of rheumatoid factor (RF) and anti-citrullinated protein antibodies (ACPA), presence of joint erosions on X-ray), disease activity (swollen and tender joint count (0–28), patient global assessment (PTGA) of disease activity and physician global assessment of disease activity (MDGA) on a 100-mm visual analogue scale (VAS; 0—best, 100—worst), erythrocyte sedimentation rate (ESR, mg/h) and C-reactive protein (CRP, mg/L)) and 28-joint disease activity score index (DAS28; 0–10) [18], Simplified Disease Activity Index (SDAI, 0–86) [19], Health Assessment Questionnaire (HAQ) for patient function with values from 0 to 3 (the higher, the worse disability) [20], EuroQol EQ-5D questionnaire for quality of life with values from − 0.59 to 1 (the higher, the better quality of life) [21], and current or previous anti-rheumatic therapies (csDMARDs, bDMARDs, tsDMARDs) and therapy with glucocorticoids (GCs). Follow-up data on disease activity, disease function and anti-rheumatic therapies are collected after 3 and 6 months, and then every 6 months for 3  years, with disease activity and anti-rheumatic therapy data collected annually thereafter.

Ethics approval for ATTRA was granted by the Czech Multicentre Research Ethics Committee, no. 201611 S300, and Institutional Ethics Committee of Institute of Rheumatology, Prague, Czech Republic, no. 10113/2016. No additional ethical approval was required for the current analysis. All subjects provided their written consent for the collection and storage of data before participation. All procedures were performed following the Declaration of Helsinki.

### Study population

In this study, we included all bio-naive adult patients diagnosed with RA starting bDMARDs/tsDMARDs within a period from 1 January 2012 to 31 January 2017 with at least 1-year follow-up. Patients without available DAS28-ESR at baseline, 6-month and 12-month visit or without HAQ and EQ-5D at baseline and 12-month visit were excluded (see Supplementary Figure 1).

### Study design

**Fig. 1:**
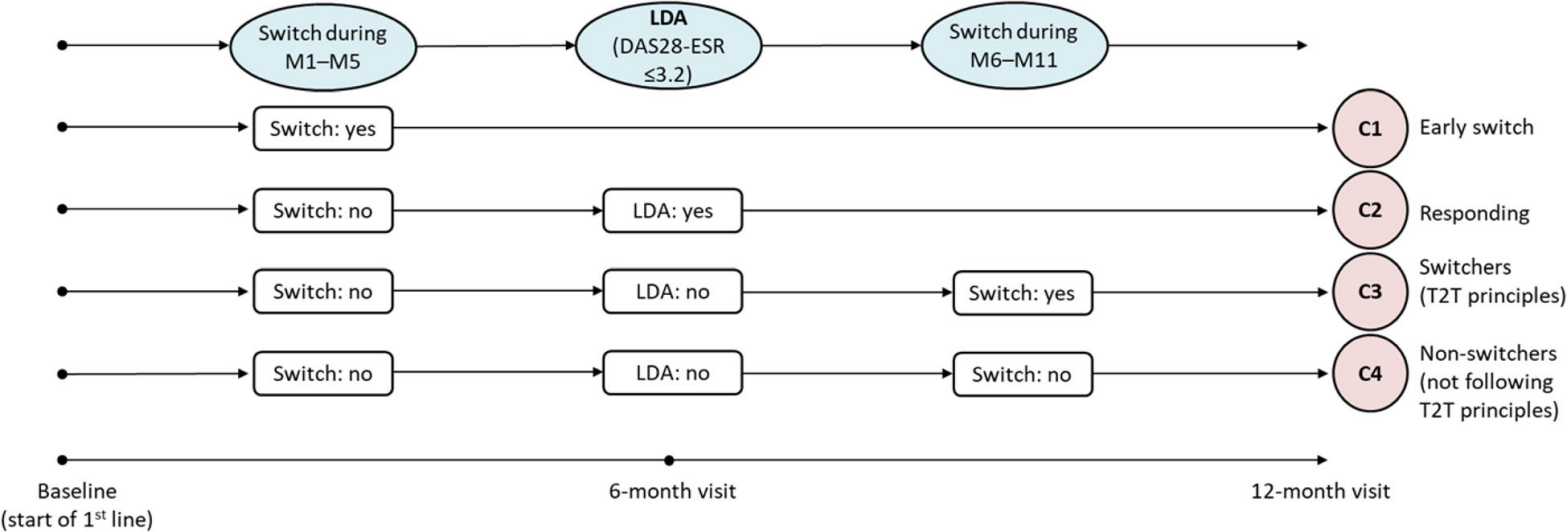
Definition of studied cohorts (C1–C4). Patients were classified based on the achievement of treatment response at the 6-month visit and based on switching to another therapy within the first year of the therapy

We divided patients into four cohorts based on a treatment result at the 6-month visit and based on switches to another therapy during the first year of the treatment (Fig. 1). First, we evaluated whether patients switched to another therapy within the first 5 months of the treatment. Next, we assessed if patients reached remission or low disease activity at the 6-month visit (defined as DAS28-ESR ≤ 3.2). Finally, we checked whether patients changed the therapy within months 6–11 provided they did not achieve the treatment target. Cohort C1 includes patients that changed bDMARD/tsDMARD therapy during the first months (usually at 3-month visit) before evaluating treatment response at the 6-month visit. These patients were either not responding to the treatment at all, or were not tolerating the treatment (e.g. side effects) within the first months of the first-line therapy. Cohort C2 consists of patients ideally responding to the treatment because they achieved the treatment target after 6 months of therapy without a need to switch. Cohort C3 comprises the group of patients not responding to the treatment, because they did not achieve the treatment target after the first 6 months of therapy. Following T2T principles, they switched to a different treatment. The last cohort C4 is represented by patients not responding to the treatment since they did not achieve the treatment target (similarly to the C3 cohort). Regardless of T2T principles, they continued with the same treatment.

### Objectives and outcome measures

The primary objective of this study was to compare odds for the achievement of REM or at least LDA after 1 year of the treatment between patients following and not following the T2T strategy (C3 vs C4). We assessed disease activity DAS28-ESR index; specifically, LDA was defined as DAS28-ESR ≤ 3.2 and REM was defined as DAS28-ESR < 2.6. In terms of the secondary outcomes, we compared treatment results based on DAS28-ESR after 12 months between all studied cohorts. The proportion of patients with remission (REM; DAS28-ESR < 2.6), low disease activity (LDA; 2.6 ≤ DAS28-ESR ≤ 3.2), moderate disease activity (MDA; 3.2 < DAS28-ESR ≤ 5.1) and high disease activity (HDA; DAS28-ESR > 5.1) at baseline and 12-month visit were compared across the studied cohorts C1–C4. Next, we compared changes in parameters related to disease activity (DAS28-ESR, SDAI, tender and swollen joint count, CRP, ESR, PTGA, MDGA) and quality of life (HAQ-DI, EQ-5D) after 6 and 12 months of the bDMARD/tsDMARD treatment between cohorts C3 and C4.

### Statistical methods

Descriptive summary of patients’ demographic and treatment characteristics and disease activity measurements was performed for all four studied cohorts C1–C4. For continuous variables, we calculated the median with interquartile range (IQR, 25th–75th percentiles). For a description of categorical variables, we used absolute and relative frequencies (i.e. percentages). To test differences between two patients’ groups, we performed the non-parametric Mann-Whitney *U* test for continuous variables and Pearson’s chi-squared test for categorical variables. The magnitude of changes in parameters over two visits was tested through the paired Wilcoxon test. For all tests, *p* values < 0.05 were considered to be statistically significant. We did not impute missing data in this analysis and performed a complete-case analysis instead. The percentage of missing data in outcome variables (i.e. DAS28-ESR, HAQ and EQ-5D at baseline, 6 and 12 months) was relatively small; we excluded 1.8% of patients in total.

We used propensity score matching to match patients not switching to another therapy after not reaching treatment target at the 6-month visit (C4) to patients switching to a different treatment after not reaching treatment target (C3). For matching, we performed logistic regression with outcome variable C3 (= 1) vs C4 (= 0) and covariates: gender, age at the start of first-line therapy, disease duration, number of previous csDMARDs, glucocorticoids in previous therapy, swollen joint count, tender joint count, PTGA, ESR, CRP, HAQ, RF positivity, presence of comorbidities, smoking, csDMARDs in concomitant therapy and glucocorticoids in previous therapy. We chose the matching ratio 1:1 and set the calliper to a value 0.2. We used matching to make both groups comparable in characteristics at the 6-month visit and to minimise confounding by other factors in the evaluation of achieving REM/LDA at the 12-month visit. After we carried out propensity score matching, we employed binary logistic regression to determine the odds for reaching REM/LDA at the 12-month visit in cohorts C3 and C4. We did all descriptive statistics and testing using IBM SPSS Statistics 25.0. The propensity score model was performed in R (version 3.5.3).

## Results

### Patients’ characteristics at baseline

In total, we included 1275 patients fulfilling the inclusion criteria into the analysis (see Supplementary Figure 1). Cohort C1 was represented by 62 (4.9%) patients, C2 consisted of 598 (46.9%) patients, C3 included 124 (9.7%) patients and 491 (38.5%) patients belonged to C4 subgroup (see Supplementary Figure 1). The most frequently administered drug was bio-original adalimumab (ranging from 27.4 to 40.3% in studied cohorts), bio-original etanercept (from 10.5 to 32.3%) and golimumab (from 6.5 to 15.5%). Tofacitinib as the only Janus kinase inhibitor administered in analysed patients was present only in one patient from C2 and one patient from C4. Out of 61 patients from C1 treated with TNFi in the first line, 42 (68.9%) switched before the 6-month visit to another TNFi, 11 (18.0%) switched to an interleukin-6 inhibitor (tocilizumab or sarilumab), 7 (11.5%) switched to abatacept and 1 (1.6%) switched to rituximab. One patient from C1 who was treated with tocilizumab in the first line switched to anakinra. Out of 120 patients from the C3 cohort that were treated with TNFi, 72 (60.0%) patients switched after the 6 months to another TNFi, 28 (23.3%) switched to an interleukin-6 drug (tocilizumab or sarilumab), 13 (10.8%) switched to abatacept and 7 (5.8%) switched to rituximab. Out of two C3 patients with tocilizumab, one switched to rituximab and the other to abatacept. Out of two C3 patients with rituximab, one switched to etanercept and the other to abatacept.

We present baseline characteristics of all four studied cohorts in Table 1. The median age at the start of the first bDMARD/tsDMARD was between 51 years (C1) and 55 years (C4 cohort). Females represented from 72.1% (C2 cohort) to 83.9% (C1 cohort) patients. Patients from all studied cohorts had high baseline disease activity according to DAS28-ESR score with median 6.4 (5.7–7.0) in C1, 5.9 (5.3–6.5) in C2, 6.2 (5.6–6.8) in C3 and 6.3 (5.8–6.8) in C4. Patients from cohorts C3 and C4 significantly differed only in age at the start of the first therapy (*p* = 0.016) and the number of previous csDMARDs (*p* = 0.025). The median age was 52.0 (44.5–61.0) years in C3 and 55.0 (48.0–63.0) years in C4. Cohorts C1 and C2 significantly differed in gender (84% vs 72% females; *p* = 0.046) and in almost all parameters related to baseline disease activity and quality of life. Patients from cohort C1 had higher disease activity than the C2 cohort according to DAS28-ESR (median 6.4 vs 5.9; *p* < 0.001), TJC (median 14 vs 12; *p* = 0.005), CRP (median 22 vs 12; *p* = 0.002), PTGA (median 78 vs 70; *p* = 0.008), MDGA (median 74 vs 65; *p* = 0.015) and worse physical function and quality of life according to HAQ-DI (median 1.8 vs 1.4; *p* = 0.001) and EQ-5D (median 0.1 vs 0.2; *p* = 0.048), respectively. Additional baseline characteristics including the presence of comorbidities, BMI, drug usage, number of concomitant csDMARDs and MTX and prednisone doses are presented in Supplementary Table 1.

**Table 1:** Baseline characteristics of patients in cohorts C1–C4 (*N* = 1275)

	C1 (*n* = 62)	C2 (*n* = 598)	C3 (*n* = 124)	C4 (*n* = 491)
Female, *n* (%)	52 (83.9%)	431 (72.1%)	102 (82.3%)	390 (79.4%)
Age at diagnosis, years, median (IQR)	44.0 (34.0–52.0)	43.5 (33.0–52.0)	45.0 (34.0–51.5)	47.0 (38.0–54.0)
Age at start of 1st line, years, median (IQR)	51.0 (42.0–58.0)	53.0 (41.0–60.0)	52.0 (44.5–61.0)	55.0 (48.0–63.0)
Disease duration, years, median (IQR)	5.6 (3.0–7.8)	6.0 (2.5–11.8)	5.0 (2.2–12.1)	6.2 (3.0–12.9)
RF positive, *n* (%)	47 (75.8%)	428 (71.6%)	92 (74.2%)	389 (79.6%)
ACPA positive, *n*/total (%)	44/61 (72.1%)	399/587 (68.0%)	91/120 (75.8%)	348/480 (72.5%)
Presence of erosions, *n*/total (%)	25/38 (65.8%)	210/295 (71.2%)	46/67 (68.7%)	22/290 (76.6%)
Currently smoking, *n*/total (%)	10/53 (18.9%)	102/504 (20.2%)	26/103 (25.2%)	93/413 (22.5%)
Number of previous csDMARDs, *n* (%)				
0	0 (0.0%)	8/592 (1.4%)	2 (1.6%)	6/484 (1.2%)
1	13 (21.0%)	226/592 (38.2%)	27 (21.8%)	131/484 (27.1%)
2	18 (29.0%)	147/592 (24.8%)	30 (24.2%)	165/484 (34.1%)
3	17 (27.4%)	112/592 (18.9%)	35 (28.2%)	113/484 (23.3%)
4+	14 (22.6%)	99/592 (16.7%)	30 (24.2%)	69/484 (14.3%)
Glucocorticoids in previous history, *n* (%)	56 (90.3%)	519/597 (86.9%)	112 (90.3%)	442 (90.0%)
Concomitant csDMARDs, *n* (%)	54 (87.1%)	549 (91.8%)	107 (86.3%)	440 (89.6%)
Concomitant MTX, *n* (%)	44 (71.0%)	454 (75.9%)	77 (62.1%)	349 (71.1%)
Concomitant GCs, *n* (%)	49 (79.0%)	446 (74.6%)	96 (77.4%)	402 (81.9%)
DAS28-ESR (0–10), median (IQR)	6.4 (5.7–7.0)	5.9 (5.3–6.5)	6.2 (5.6–6.8)	6.3 (5.8–6.8)
TJC (28 joints), median (IQR)	14.0 (11.0–19.0)	12.0 (9.0–16.0)	14.5 (9.0–19.0)	13.0 (10.0–18.0)
SJC (28 joints), median (IQR)	10.0 (7.0–13.0)	9.0 (6.0–12.0)	9.5 (6.0–12.5)	10.0 (7.0–13.0)
ESR (mm/h), median (IQR)^a^	33.5 (16.0–53.0)	28.0 (16.0–40.0)	32.0 (18.0–50.0)	34.0 (23.0–50.0)
CRP (mg/l), median (IQR)^b^	22.0 (9.4–34.0)	12.0 (5.3–23.5)	15.0 (7.9–31.0)	16.8 (8.0–33.1)
SDAI (0–86), median (IQR)^c^	40.5 (32.7–47.8)	35.6 (29.5–42.4)	39.3 (33.0–48.2)	39.0 (32.6–45.9)
PTGA (0–100), median (IQR)	78.0 (69.0–84.0)	70.0 (59.0–80.0)	75.0 (62.5–81.5)	75.0 (60.0–85.0)
MDGA (0–100), median (IQR)^d^	74.0 (60.0–80.0)	65.0 (52.0–75.0)	70.0 (58.5–80.0)	70.0 (60.0–80.0)
HAQ-DI (0–3), median (IQR)	1.8 (1.3–2.0)	1.4 (1.0–1.8)	1.8 (1.4–2.0)	1.8 (1.4–2.0)
EQ-5D (− 0.59–1), median (IQR)	0.1 (0.0–0.6)	0.2 (0.1–0.7)	0.1 (0.0–0.5)	0.1 (0.0–0.5)

*IQR* interquartile range, *RF* rheumatoid factor, *ACPA* anti-citrullinated protein, *TNFi* tumour necrosis factor inhibitor, *csDMARDs* conventional synthetic disease-modifying anti-rheumatic drugs, *MTX* methotrexate, *DAS28-ESR* 28-joint disease activity score with ESR, *TJC* tender joint count, *SJC* swollen joint count, *ESR* erythrocyte sedimentation rate, *CRP* C-reactive protein, *SDAI* Simplified Disease Activity Index, *PTGA* patient general assessment of disease activity, *MDGA* physician general assessment of disease activity, *HAQ-DI* Health Assessment Questionnaire, *EQ-5D* EuroQol 5 Dimension for measuring the quality of life

^a^*n* = 62 (C1), *n* = 573 (C2), *n* = 118 (C3), *n* = 486 (C4)

^b^*n* = 60 (C1), *n* = 576 (C2), *n* = 123 (C3), *n* = 480 (C4)

^c^*n* = 55 (C1), *n* = 560 (C2), *n* = 119 (C3), *n* = 465 (C4)

^d^*n* = 57 (C1), *n* = 582 (C2), *n* = 120 (C3), *n* = 476 (C4)

### Disease activity after 3 months in C1–C4

Disease activity according to DAS28-ESR score after 3 months of the first-line treatment in cohorts C1–C4 is shown in Supplementary Figure 2. We observed the highest proportion of patients with REM/LDA after 3 months in cohort C2 in almost 70% of patients. This result was statistically significantly higher compared to other cohorts (*p* < 0.001). There was also a statistically significant difference in the proportion of patients with REM/LDA between patients in the groups C3 and C4. While in group C3 it was 10.6% of patients, in group C4 it was 20.4% (*p* = 0.016). The median value of DAS28-ESR in group C1 corresponded to the high disease activity range; group C2 had median DAS28-ESR belonging to low disease activity; the median DAS28-ESR value in cohorts C3 and C4 fell into the category of moderate disease activity.

### Disease activity after 12 months in C1–C4

**Fig. 2:**
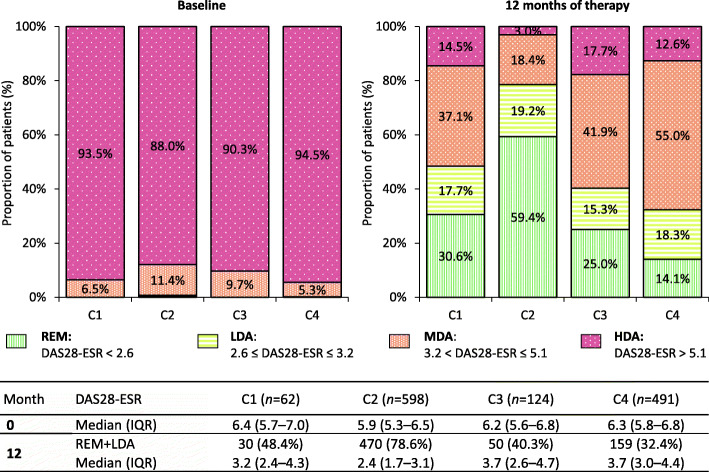
Disease activity according to DAS28-ESR at baseline and after 1 year of treatment. DAS28-ESR, 28-joint disease activity score with erythrocyte sedimentation; REM, remission; LDA, low disease activity; MDA, moderate disease activity; HDA, high disease activity; IQR, interquartile range

Comparison of disease activity according to the DAS28-ESR score after 1 year of treatment in cohorts C1–C4 is illustrated in Fig. 2. We could see the best treatment results after 12 months in group C2 with almost 79% patients with REM/LDA compared to 48% patients in group C1 (*p* < 0.001), 40% patients in group C3 (*p* < 0.001) and 32% in group C4 (*p* < 0.001). Although there was no statistically significant difference in the proportion of patients with REM/LDA between groups C3 (following T2T strategy) and C4 (not following T2T strategy) after 12 months (*p* = 0.095), we could observe slightly better results in group C3 (40% vs 32% with REM/LDA). We observed the lowest median value of DAS28-ESR in group C2, and it falls within the level of remission. The median value of DAS28-ESR in group C1 corresponded to the upper bound of low disease activity, and median DAS28-ESR values in groups C3 and C4 were within the range of moderate disease activity.

### Comparison of cohorts C3 and C4 at 6-month and 12-month visit

At the 6-month visit, patients from groups C3 and C4 differed in all tested parameters related to disease activity and quality of life (Table 2). We observed lower disease activity and better quality of life in C4. Patients from C3 and C4 did not significantly differ in concomitant therapy, but numerically more changes in dosage of glucocorticoids and methotrexate have been observed in the C3 cohort compared to the C4 cohort between M6 and M12 (see Supplementary Table 2). At the 12-month visit, patients from both groups did not significantly differ in most of the parameters related to disease activity; they only differed in PTGA (*p* = 0.044) and EQ-5D (*p* = 0.017). In terms of the magnitude of changes across the two visits, patients from C3 significantly improved in all parameters related to disease activity and quality of life (see Supplementary Table 3). Patients from C4 did not significantly improve in CRP and HAQ-DI. In the comparison of the size of changes between the two groups, patients from C3 showed better results (i.e. more significant improvements) in all tested parameters (see Supplementary Table 3).

**Table 2:** Comparison of parameters related to disease activity, quality of life and concomitant therapy between C3 and C4 cohorts at the 6-month and 12-month visit

	6 months			12 months		
	C3 (*n* = 124)	C4 (*n* = 491)	*p* value	C3 (*n* = 124)	C4 (*n* = 491)	*p* value
DAS28-ESR (0–10)	5.4 (4.6–6.3)	4.0 (3.5–4.5)	< **0.001**	3.7 (2.6–4.7)	3.7 (3.0–4.4)	0.710
TJC (28 joints)	9.0 (4.0–14.0)	3.0 (2.0–5.0)	< **0.001**	3.0 (1.0–7.0)	2.0 (1.0–5.0)	0.490
SJC (28 joints)	6.0 (2.0–9.5)	2.0 (1.0–4.0)	< **0.001**	2.0 (0.0–4.0)	2.0 (0.0–3.0)	0.498
ESR (mm/h)	28.0 (16.5–46.5)	22.0 (13.0–33.0)	< **0.001**	16.5 (6.5–32.0)	19.0 (11.0–30.5)	0.052
CRP (mg/l)	15.0 (7.9–28.9)	5.7 (2.5–13.7)	< **0.001**	4.7 (1.6–17.0)	5.0 (2.3–11.3)	0.766
SDAI (0–86)	30.2 (19.7–39.5)	13.9 (10.7–18.3)	< **0.001**	13.8 (8.0–20.9)	11.3 (7.7–17.4)	0.093
PTGA (0–100)	61.0 (50.0–75.0)	40.0 (26.0–50.0)	< **0.001**	36.0 (25.0–60.0)	33.0 (20.0–50.0)	**0.044**
MDGA (0–100)	58.0 (40.0–70.0)	30.0 (20.0–40.0)	< **0.001**	25.0 (15.0–45.0)	25.0 (15.0–40.0)	0.812
HAQ-DI (0–3)	1.5 (1.1–1.9)	1.3 (0.9–1.6)	< **0.001**	1.3 (0.9–1.9)	1.3 (0.9–1.6)	0.140
EQ-5D (− 0.59–1)	0.2 (0.1–0.7)	0.7 (0.5–0.7)	< **0.001**	0.6 (0.1–0.7)	0.7 (0.5–0.8)	**0.017**
Concomitant csDMARDs	98 (79.0%)	414 (84.3%)	0.159	94 (75.8%)	407 (82.9%)	0.070
Concomitant MTX	79 (63.7%)	341 (69.5%)	0.220	77 (62.1%)	332 (67.6%)	0.245
Concomitant GCs	95 (76.6%)	374 (76.2%)	0.918	92 (74.2%)	370 (75.4%)	0.789

Continuous variables are described through the median (interquartile range); categorical variables are characterised by *n* (%)

*DAS28-ESR* 28-joint disease activity score with ESR, *TJC* tender joint count, *SJC* swollen joint count, *ESR* erythrocyte sedimentation rate, *CRP* C-reactive protein, *SDAI* Simplified Disease Activity Index, *PTGA* patient general assessment of disease activity, *MDGA* physician general assessment of disease activity, *HAQ-DI* Health Assessment Questionnaire, *EQ-5D* EuroQol 5 Dimension for measuring the quality of life, *csDMARDs* conventional synthetic disease-modifying anti-rheumatic drugs, *MTX* methotrexate, *GCs* glucocorticoids

### Odds for treatment target in C3 vs C4 at the 12-month visit

We employed propensity score matching to reduce selection bias by adjusting for potential confounding factors at the 6-month visit. We show a description of patients’ characteristics at the 6-month visit after using propensity score matching in Table 3. Both groups included 75 patients after the matching. Density plots of propensity score before and after matching are displayed in Supplementary Figure 3. Patients did not differ anymore in parameters related to disease activity and quality of life (see Table 3). The most frequently administered drugs at the 12-month visit were tocilizumab (27%), certolizumab (17%), abatacept (15%) and etanercept (12%) in C3. Patients from C4 were most frequently treated with adalimumab (35%), etanercept (21%), golimumab (16%) and certolizumab (13%). To compare odds for reaching treatment target at the 12-month visit in patients following the T2T principle at 6-month visit (C3) vs patients staying on the first treatment (C4), we employed a logistic regression model with outcome DAS28-ESR ≤ 3.2. Patients following the T2T principle (C3) showed 2.8 (CI 1.4–5.8) times higher odds for reaching at least LDA at the 12-month visit (*p* = 0.005) compared to patients not following the T2T principle (C4). In group C3, 41% of patients achieved at least REM/LDA at the 12-month visit, while in group C4, it was 20% (see Supplementary Table 4).

**Table 3:** Description of patients from C3 and C4 cohorts at the 6-month visit after applying propensity score matching

	C3 (*n* = 75)	C4 (*n* = 75)	*p* value
Female, *n* (%)*	60 (80.0%)	61 (81.3%)	0.836
Age at diagnosis, years, median (IQR)	45.0 (36.0–53.0)	45.0 (37.0–53.0)	0.678
Age at start of 1st line, years, median (IQR)*	52.0 (45.0–61.0)	55.0 (44.0–61.0)	0.811
Disease duration, years, median (IQR)*	5.0 (2.4–12.7)	5.8 (3.0–13.1)	0.937
RF positive, *n* (%)*	60 (80.0%)	54 (72.0%)	0.251
Presence of comorbidities, *n* (%)*	54 (72.0%)	55 (73.3%)	0.855
Currently smoking, *n* (%)*	21 (28.0%)	21 (28.0%)	1.000
Number of previous csDMARDs, *n* (%)*			
0	2 (2.7%)	0 (0.0%)	0.230
1	16 (21.3%)	15 (20.0%)	
2	20 (26.7%)	28 (37.3%)	
3	17 (22.7%)	20 (26.7%)	
4+	20 (26.7%)	12 (16.0%)	
Glucocorticoids in previous history, *n* (%)*	67 (89.3%)	66 (88.0%)	0.797
Concomitant csDMARDs, *n* (%)*	61 (81.3%)	63 (84.0%)	0.666
Concomitant GCs, *n* (%)*	56 (74.7%)	55 (73.3%)	0.852
DAS28-ESR (0–10), median (IQR)	5.0 (4.2–5.9)	5.0 (4.1–5.7)	0.717
TJC (28 joints), median (IQR)*	8.0 (4.0–12.0)	6.0 (3.0–11.0)	0.677
SJC (28 joints), median (IQR)*	4.0 (2.0–8.0)	4.0 (2.0–7.0)	0.973
ESR (mm/h), median (IQR)*	27.0 (15.0–37.0)	25.0 (12.0–41.0)	0.844
CRP (mg/l), median (IQR)*	15.0 (8.0–22.2)	8.4 (3.5–25.7)	0.090
SDAI (0–86), median (IQR)	25.5 (15.6–34.9)	22.7 (16.1–30.9)	0.531
PTGA (0–100), median (IQR)*	60.0 (40.0–71.0)	50.0 (40.0–71.0)	0.519
MDGA (0–100), median (IQR)	55.0 (35.0–70.0)	45.0 (30.0–60.0)	0.059
HAQ-DI (0–3), median (IQR)*	1.5 (1.1–1.9)	1.5 (1.1–1.9)	0.877
EQ-5D (− 0.59–1), median (IQR)^a^	0.2 (0.1–0.7)	0.6 (0.1–0.7)	0.290

These parameters were included in the propensity score model

*IQR* interquartile range, *RF* rheumatoid factor, *csDMARDs* conventional synthetic disease-modifying anti-rheumatic drugs, *GCs* glucocorticoids, *DAS28-ESR* 28-joint disease activity score with ESR, *TJC* tender joint count, *SJC* swollen joint count, *ESR* erythrocyte sedimentation rate, *CRP* C-reactive protein, *SDAI* Simplified Disease Activity Index, *PTGA* patient general assessment of disease activity, *MDGA* physician general assessment of disease activity, *HAQ-DI* Health Assessment Questionnaire, *EQ-5D* EuroQol 5 Dimension for measuring the quality of life

^a^*n* = 74 (C3), *n* = 75 (C4)

## Discussion

In this prospective observational cohort study from real clinical practice in the Czech Republic, we have shown that following the T2T strategy and switching the targeted drug to another therapy after not reaching REM/LDA at the 6-month visit increases the chance of achieving REM/LDA at the 12-month visit as opposed to patients not following the treatment target. This finding support results from previous studies showing that T2T is efficient in daily clinical practice. Our study also provided a summary of four different courses of treatment management during the first year of bDMARD/tsDMARD therapy. We created four patients’ cohorts based on switching the treatment and based on reaching a treatment target at 6 months. We described all four patients’ groups at baseline and compared their treatment results after 1 year of treatment. Furthermore, we evaluated disease activity and quality of life at 6 months in groups C3 and C4 and compared the sizes of changes from the 6-month to the 12-month visit. We observed that patients not following the T2T at the 6-month visit (C4) had lower disease activity and better quality of life at 6 months than patients following T2T and switching to another therapy after not reaching the treatment target (C3). However, patients following the T2T strategy showed a more significant improvement both in disease activity and quality of life within the period from the 6-month visit to the 12-month visit. Patients from cohort C3 also had a higher rate of REM/LDA at 12 months in comparison with C4 (though not statistically significant; *p* = 0.095).

A similar study investigated whether a tight control treatment strategy (i.e. optimising treatment by measurement of disease activity in order to make treatment adjustments to reach a predefined target LDA/REM) in early RA is more effective than treatment according to usual care in reaching REM (DAS28 < 2.6) after 1 year [4]. They compared two distinct early RA cohorts from two different regions in the Netherlands: the usual care cohort and the ‘tight control’ cohort. The OR adjusted for baseline DAS28 was 3.1 (95% CI 1.8–5.2). Therefore, patients treated according to tight control had approximately three times higher odds to reach REM at 1 year after the baseline. This result is very similar to the OR obtained in our study, though we evaluated achievement of LDA/REM instead. In another similar study, Norwegian authors compared patients following a T2T strategy (2010–2015) with patients from the pre-T2T cohort (2006–2009) following routine care [13]. They assessed the 2-year effect on disease activity and health-related quality of life and showed significantly higher odds (multivariable OR 1.89, 95% CI 1.33–2.68) for SDAI remission (≤ 3.3) in patients following a T2T strategy. Within secondary outcomes, they also evaluated REM, according to DAS28 (OR 2.15, 95% CI 1.51–3.06).

A Dutch study investigated the 3-year results of a protocolised T2T strategy in daily clinical practice [16]. The authors found out that T2T leads to high remission rates, improved physical function and quality of life, and limited radiographic damage after 3 years in daily clinical practice. In another study from the Netherlands, the authors described a 5-year continuous application of a T2T strategy in patients with early RA in daily clinical practice and confirmed the favourable disease- and patient-related outcomes [12]. A longitudinal study of RA patients from 10 countries (RA BIODAM) investigated whether following a T2T strategy in daily clinical practice leads to more patients meeting REM [14]. Application of T2T every 3 months did not yield a higher likelihood of REM according to DAS44 and DAS28 3 months later, but sustained T2T (i.e. T2T followed in at least two consecutive visits) resulted in an increased likelihood of achieving DAS44 REM (OR 1.19, 95% CI 1.03–1.39).

Our study has shown that a substantial number of patients did not follow the T2T strategy and continued with the same treatment after not reaching the treatment target within 6 months. This finding is probably not unique for the Czech Republic. Others have also shown that the T2T strategy is underused in real clinical practice; e.g. in the analysis from the Corrona RA registry, a considerable proportion of patients continued without changing/accelerating treatment despite not reaching an adequate response to the initial TNF inhibitor therapy at 6 and 12 months [22].

Although the present study has a limitation of the absence of randomisation, we have partially overcome this problem by employing the propensity score matching at the 6-month visit. Thus, we have minimised confounding by other factors, and we obtained the effect of following/not following the T2T principle in the evaluation of REM/LDA at the 12-month visit. A possible limitation of this study could be an absence of monitoring treatment intensification through increased dosages. Further, our study only concerned the first-line bDMARD/tsDMARD therapy. Thus, evaluating of implementation of the T2T strategy within subsequent lines of therapy could be a possible subject for future studies.

## Conclusion

In conclusion, the application of T2T principles and switching to another bDMARD/tsDMARD after not reaching REM/LDA within the first 6 months of bDMARD/tsDMARD treatment leads to a higher probability of achieving REM/LDA in RA patients at the 12-month visit. In this study, the T2T strategy showed superiority over traditional routine care in daily clinical practice.

## Supplementary Information

**Additional file 1: Supplementary Table 1.** Additional baseline characteristics of patients in cohorts C1–C4 (*N*=1275).

**Additional file 2: Supplementary Table 2.** Changes in comedication with glucocorticoids and methotrexate during the first year in cohorts C1–C4.

**Additional file 3: Supplementary Table 3.** Comparison of differences in parameters from month 6 to 12 between cohort C3 and C4.

**Additional file 4: Supplementary Table 4.** Results of logistic regression with outcome DAS28-ESR ≤ 3.2 for C3 vs C4.

**Additional file 5: Supplementary Figure 1.** Flow chart showing individual steps to final dataset (a) and division into four cohorts (b).

**Additional file 6: Supplementary Figure 2.** Disease activity according to DAS28-ESR after three months of treatment.

**Additional file 7: Supplementary Figure 3.** Propensity score densities of cohorts C3 and C4 before and after matching.

## Data Availability

The datasets analysed during the current study are available from the corresponding author on reasonable request. Requests will be considered by the Czech Rheumatological Society.

## Abbreviations

ACPA: Anti-citrullinated protein antibodies
bDMARDs: Biological disease-modifying anti-rheumatic drugs
CI: Confidence interval
CRP: C-reactive protein
csDMARDs: Conventional synthetic disease-modifying anti-rheumatic drugs
CZ: Czech Republic
DAS28-ESR: 28-joint disease activity score using the erythrocyte sedimentation rate
DMARD: Disease-modifying anti-rheumatic drug
EQ-5D: EuroQol-5 Dimensions
ESR: Erythrocyte sedimentation rate
EULAR: European League Against Rheumatism
GCs: Glucocorticoids
HAQ-DI: Health Assessment Questionnaire Disability Index
HDA: High disease activity
IQR: Interquartile range
LDA: Low disease activity
MDA: Moderate disease activity
MDGA: Physician global assessment of disease activity
MTX: Methotrexate
OR: Odds ratio
PTGA: Patient global assessment of disease activity
RA: Rheumatoid arthritis
REM: Remission
RF: Rheumatoid factor
SDAI: Simplified disease activity index
T2T: Treat-to-target
TNFi: Tumour necrosis factor inhibitor
tsDMARDs: Targeted disease-modifying anti-rheumatic drugs
TT: Targeted therapy
VAS: Visual analogue scale
