# Supplementary material for: Switching first-line targeted therapy after not reaching low disease activity within 6 months is superior to conservative approach: a propensity score-matched analysis from the ATTRA registry
Source: Arthritis Res Ther. 2021 Jan 6;23:11. doi: 10.1186/s13075-020-02393-8 (PMC7789592; doi:10.1186/s13075-020-02393-8)
Supplement: Supplementary file 1 — Additional file 1: Supplementary Table 1. Additional baseline characteristics of patients in cohorts C1–C4 (N=1275). [file 13075_2020_2393_MOESM1_ESM.docx]

**Supplementary Table 1** Additional baseline characteristics of patients in cohorts C1–C4 (N=1275)

|  | C1 (*n*=62) | C2 (*n*=598) | C3 (*n*=124) | C4 (*n*=491) |
| --- | --- | --- | --- | --- |
| Presence of comorbidities, *n* (%) | 44 (71.0%) | 364 (61.0%) | 77 (62.1%) | 334 (68.0%) |
| - gastrointestinal disorders, *n* (%) | 10 (16.1%) | 40 (6.7%) | 12 (9.7%) | 51 (10.4%) |
| - peptic ulcer | 5 (50.0%) | 14 (35.0%) | 2 (16.7%) | 22 (43.1%) |
| - hepatopathy | 4 (40.0%) | 16 (40.0%) | 5 (41.7%) | 23 (45.1%) |
| - IBD | 1 (10.0%) | 6 (15.0%) | 1 (8.3%) | 4 (7.8%) |
| - infectious disease, *n* (%) | 5 (8.3%) | 26 (4.4%) | 7 (5.7%) | 24 (5.0%) |
| - hepatitis B | 1 (20.0%) | 8 (30.8%) | 0 (0.0%) | 5 (20.8%) |
| - hepatitis C | 0 (0.0%) | 2 (7.7%) | 2 (28.6%) | 0 (0.0%) |
| - latent tuberculosis | 2 (40.0%) | 12 (46.2%) | 5 (71.4%) | 14 (58.3%) |
| - diverticulitis | 0 (0.0%) | 2 (7.7%) | 0 (0.0%) | 3 (12.5%) |
| - metabolic disorder, *n* (%) | 11 (18.3%) | 126 (21.2%) | 17 (13.9%) | 112 (23.1%) |
| - hyperlipidemia | 8 (72.7%) | 91 (72.2%) | 12 (70.6%) | 79 (70.5%) |
| - diabetes mellitus | 4 (36.4%) | 42 (33.3%) | 6 (35.3%) | 44 (39.3%) |
| - malignancy, *n* (%) | 1 (1.7%) | 11 (1.9%) | 4 (3.3%) | 16 (3.3%) |
| - lymphoma/lymphoprolif. disorder | 1 (100.0%) | 1 (9.1%) | 0 (0.0%) | 1 (6.3%) |
| - scin cancer - melanoma | 0 (0.0%) | 1 (9.1%) | 1 (25.0%) | 0 (0.0%) |
| - scin cancer - basal cell carcinoma | 0 (0.0%) | 0 (0.0%) | 0 (0.0%) | 1 (6.3%) |
| - solid tumours | 0 (0.0%) | 6 (54.5%) | 3 (75.0%) | 14 (87.5%) |
| - neuropsychiatric disease, *n* (%) | 1 (1.7%) | 30 (5.1%) | 7 (5.7%) | 29 (6.0%) |
| - demyelinating disease | 0 (0.0%) | 2 (6.7%) | 0 (0.0%) | 1 (3.4%) |
| - depression | 1 (100.0%) | 23 (76.7%) | 6 (85.7%) | 26 (89.7%) |
| - cardiovascular disease, *n* (%) | 22 (36.7%) | 211 (35.8%) | 48 (39.0%) | 194 (40.0%) |
| - hypertension | 16 (72.7%) | 190 (90.0%) | 45 (93.8%) | 179 (92.3%) |
| - heart attack | 0 (0.0%) | 5 (2.4%) | 0 (0.0%) | 5 (2.6%) |
| - ischemic heart disease | 2 (9.1%) | 10 (4.7%) | 2 (4.2%) | 20 (10.3%) |
| - heart failure | 0 (0.0%) | 1 (0.5%) | 0 (0.0%) | 1 (0.5%) |
| - stroke/transient ischemic attack | 0 (0.0%) | 4 (1.9%) | 1 (2.1%) | 5 (2.6%) |
| - deep vein thrombosis/pulmonary embolism | 3 (13.6%) | 12 (5.7%) | 4 (8.3%) | 15 (7.7%) |
| - other comorbidities, *n* (%) | 28 (46.7%) | 206 (35.3%) | 40 (33.3%) | 189 (39.1%) |
| - asthma/COPD/emphysema | 7 (25.0%) | 30 (14.6%) | 9 (22.5%) | 25 (13.2%) |
| - psoriasis | 0 (0.0%) | 8 (3.9%) | 3 (7.5%) | 12 (6.3%) |
| - neuropathy | 1 (3.6%) | 5 (2.4%) | 0 (0.0%) | 5 (2.6%) |
| - osteoporosis | 0 (0.0%) | 0 (0.0%) | 0 (0.0%) | 1 (0.5%) |
| - other | 25 (89.3%) | 177 (85.9%) | 31 (77.5%) | 160 (84.7%) |
| Number of comorbidities, n |  |  |  |  |
| Presence of 1 comorbidity, *n* (%) | 23 (52.3%) | 173 (47.5%) | 42 (54.5%) | 147 (44.0%) |
| Presence of 2 comorbidities, *n* (%) | 10 (22.7%) | 114 (31.3%) | 20 (26.0%) | 99 (29.6%) |
| Presence of 3+ comorbidities, *n* (%) | 11 (25.0%) | 77 (21.2%) | 15 (19.5%) | 88 (26.3%) |
| BMI, median (IQR) | 24.9 (23.1–28.1) | 25.6 (22.6–29.4) | 25.5 (22.9–30.4) | 26.1 (22.8–30.1) |
| Underweight (BMI<18.5), *n*/total (%) | 1/58 (1.7%) | 11/576 (1.9%) | 3/121 (2.5%) | 13/475 (2.7%) |
| Normal weight (BMI 18.5–24.9), *n*/total (%) | 29/58 (50.0%) | 251/576 (43.6%) | 54/121 (44.6%) | 185/475 (38.9%) |
| Overweight (BMI 25–29.9), *n*/total (%) | 20/58 (34.5%) | 192/576 (33.3%) | 32/121 (26.4%) | 151/475 (31.8%) |
| Obesity (BMI≥30), *n*/total (%) | 8/58 (13.8%) | 122/576 (21.2%) | 32/121 (26.4%) | 126/475 (26.5%) |
| TNFi, *n* (%) | 61 (98.4%) | 556 (93.0%) | 120 (96.8%) | 457 (93.1%) |
| Tocilizumab, *n* (%) | 1 (1.6%) | 29 (4.8%) | 2 (1.6%) | 5 (1.0%) |
| Rituximab, *n* (%) | 0 (0.0%) | 8 (1.3%) | 2 (1.6%) | 24 (4.9%) |
| Abatacept, *n* (%) | 0 (0.0%) | 4 (0.7%) | 0 (0.0%) | 4 (0.8%) |
| Tofacitinib, *n* (%) | 0 (0.0%) | 1 (0.2%) | 0 (0.0%) | 1 (0.2%) |
| Concomitant csDMARDs, *n* (%) | 54 (87.1%) | 549 (91.8%) | 107 (86.3%) | 440 (89.6%) |
| 1 csDMARD in conc. therapy, *n* (%) | 45 (83.3%) | 470 (85.6%) | 88 (82.2%) | 340 (77.3%) |
| 2 csDMARDs in conc. therapy, *n* (%) | 7 (13.0%) | 67 (12.2%) | 16 (15.0%) | 96 (21.8%) |
| 3 csDMARDs in conc. therapy, *n* (%) | 2 (3.7%) | 12 (2.2%) | 3 (2.8%) | 4 (0.9%) |
| Concomitant MTX, *n* (%) | 44 (71.0%) | 454 (75.9%) | 77 (62.1%) | 349 (71.1%) |
| MTX dose, mg/week, median (IQR) | 15.0 (10.0; 20.0) | 15.0 (15.0; 20.0) | 20.0 (10.0; 20.0) | 15.0 (12.5; 20.0) |
| Concomitant GCs, *n* (%) | 49 (79.0%) | 446 (74.6%) | 96 (77.4%) | 402 (81.9%) |
| Prednisone dose, mg/day, median (IQR) | 7.5 (5.0; 10.0) | 5.0 (5.0; 10.0) | 7.5 (5.0; 10.0) | 7.9 (6.1; 7.5) |

*IQR* interquartile range; *BMI* body mass index; *TNFi* tumour necrosis factor inhibitor (adalimumab, certolizumab, etanercept, golimumab, infliximab); *csDMARDs* conventional synthetic disease-modifying anti-rheumatic drugs; *MTX* methotrexate; *GCs* glucocorticoids; *IBD* nonspecific inflammatory bowel disease
