# Supplementary material for: Switching first-line targeted therapy after not reaching low disease activity within 6 months is superior to conservative approach: a propensity score-matched analysis from the ATTRA registry
Source: Arthritis Res Ther. 2021 Jan 6;23:11. doi: 10.1186/s13075-020-02393-8 (PMC7789592; doi:10.1186/s13075-020-02393-8)
Supplement: Supplementary file 2 — Additional file 2: Supplementary Table 2. Changes in comedication with glucocorticoids and methotrexate during the first year in cohorts C1–C4. [file 13075_2020_2393_MOESM2_ESM.docx]

**Supplementary Table 2** Changes in comedication with glucocorticoids and methotrexate during the first year in cohorts C1–C4

| Glucocorticoids (GCs) | C1 | C2 | C3 | C4 |
| --- | --- | --- | --- | --- |
| Change in comedication with GCs in M1–M5 (yes → no or no → yes) | 17.0% | 11.2% | 7.1% | 7.2% |
| Change in comedication with GCs in M6–M12 (yes →no or no → yes) | 9.1% | 8.5% | 17.1% | 5.7% |
| No change in M1–M12 (yes → yes or no → no) | 73.9% | 80.3% | 75.8% | 87.1% |
| Methotrexate (MTX) | C1 | C2 | C3 | C4 |
| Change in comedication with MTX in M1–M5 (yes → no or no → yes) | 15.4% | 5.0% | 2.7% | 2.9% |
| Change in comedication with MTX in M6–M12 (yes →no or no → yes) | 4.5% | 2.8% | 7.3% | 3.2% |
| No change in M1–M12 (yes → yes or no → no) | 80.1% | 92.1% | 90.1% | 93.8% |

*GCs* glucocorticoids; *M* month; *MTX* methotrexate

Percentages of patients changing the comedication with GCs and MTX (in the sense of starting or discontinuing the comedication compared to the situation at baseline) within the studied period are presented in the table.
