# Supplementary material for: Switching first-line targeted therapy after not reaching low disease activity within 6 months is superior to conservative approach: a propensity score-matched analysis from the ATTRA registry
Source: Arthritis Res Ther. 2021 Jan 6;23:11. doi: 10.1186/s13075-020-02393-8 (PMC7789592; doi:10.1186/s13075-020-02393-8)
Supplement: Supplementary file 3 — Additional file 3: Supplementary Table 3. Comparison of differences in parameters from month 6 to 12 between cohort C3 and C4. [file 13075_2020_2393_MOESM3_ESM.docx]

**Supplementary Table 3** Comparison of differences in parameters from month 6 to 12 between cohort C3 and C4

|  | C3 (*n*=124) | P-value* | C4 (*n*=491) | P-value* | P-value† |
| --- | --- | --- | --- | --- | --- |
| DAS28-ESR | -1.60 (-2.81; -0.49) | <**0.001** | -0.35 (-1.00; 0.28) | <**0.001** | <**0.001** |
| TJC (28 joints) | -5.00 (-10.00; -1.00) | <**0.001** | -1.00 (-2.00; 1.00) | <**0.001** | <**0.001** |
| SJC (28 joints) | -3.00 (-7.00; 0.00) | <**0.001** | 0.00 (-1.00; 0.00) | <**0.001** | <**0.001** |
| ESR (mm/h) | -9.50 (-22.00; 0.00) | <**0.001** | -1.00 (-10.00; 6.00) | **0.015** | <**0.001** |
| CRP (mg/l) | -7.31 (-16.15; 1.39) | <**0.001** | -0.20 (-3.50; 3.00) | 0.052 | <**0.001** |
| SDAI (0–86) | -15.34 (-25.13; -4.85) | <**0.001** | -2.23 (-5.83; 1.93) | <**0.001** | <**0.001** |
| PTGA (0–100)d | -16.00 (-41.50; 0.00) | <**0.001** | -5.00 (-15.00; 7.00) | <**0.001** | <**0.001** |
| MDGA (0–100) | -26.00 (-45.00; -3.50) | <**0.001** | -3.00 (-15.00; 6.00) | <**0.001** | <**0.001** |
| HAQ-DI (0–3)^d^ | -0.24 (-0.38; 0.00) | <**0.001** | 0.00 (-0.25; 0.13) | 0.059 | <**0.001** |
| EQ-5D (-0.59–1) | 0.04 (0.00; 0.53) | <**0.001** | 0.00 (-0.05; 0.07) | **0.032** | <**0.001** |

*DAS28-ESR* 28-joint disease activity score with ESR; *TJC* tender joint count; *SJC* swollen joint count; *ESR* erythrocyte sedimentation rate; *CRP* C-reactive protein; *SDAI* Simplified Disease Activity Index; *PTGA* patient general assessment of disease activity; *MDGA* physician general assessment of disease activity; *HAQ‑DI* Health Assessment Questionnaire; *EQ-5D* EuroQol 5 Dimension for measuring the quality of life

Medians of differences between 6-month and 12-month visit with interquartile ranges are presented.

* We were testing the hypothesis that the differences are equal to zero through Wilcoxon paired test.

† We were testing the hypothesis of equality of medians of differences between groups C3 and C4 (Mann-Whitney test).
