# Supplementary material for: Switching first-line targeted therapy after not reaching low disease activity within 6 months is superior to conservative approach: a propensity score-matched analysis from the ATTRA registry
Source: Arthritis Res Ther. 2021 Jan 6;23:11. doi: 10.1186/s13075-020-02393-8 (PMC7789592; doi:10.1186/s13075-020-02393-8)
Supplement: Supplementary file 4 — Additional file 4: Supplementary Table 4. Results of logistic regression with outcome DAS28-ESR ≤ 3.2 for C3 vs C4. [file 13075_2020_2393_MOESM4_ESM.docx]

**Supplementary Table 4** Results of logistic regression with outcome DAS28-ESR ≤ 3.2 for C3 vs C4

| Cohort | OR (95% CI) | P-value | n |
| --- | --- | --- | --- |
| C3 (vs C4) | 2.82 (1.36–5.84) | **0.005** | 150 |

OR odds ratio; CI confidence interval
