# Supplementary material for: Switching first-line targeted therapy after not reaching low disease activity within 6 months is superior to conservative approach: a propensity score-matched analysis from the ATTRA registry
Source: Arthritis Res Ther. 2021 Jan 6;23:11. doi: 10.1186/s13075-020-02393-8 (PMC7789592; doi:10.1186/s13075-020-02393-8)
Supplement: Supplementary file 5 — Additional file 5: Supplementary Figure 1. Flow chart showing individual steps to final dataset (a) and division into four cohorts (b). [file 13075_2020_2393_MOESM5_ESM.docx]

**Supplementary Figure 1** Flow chart showing individual steps to final dataset (a) and division into four cohorts (b)
