# Supplementary material for: Switching first-line targeted therapy after not reaching low disease activity within 6 months is superior to conservative approach: a propensity score-matched analysis from the ATTRA registry
Source: Arthritis Res Ther. 2021 Jan 6;23:11. doi: 10.1186/s13075-020-02393-8 (PMC7789592; doi:10.1186/s13075-020-02393-8)
Supplement: Supplementary file 6 — Additional file 6: Supplementary Figure 2. Disease activity according to DAS28-ESR after three months of treatment. [file 13075_2020_2393_MOESM6_ESM.docx]

**Supplementary Figure 2** Disease activity according to DAS28-ESR after three months of treatment

DAS28-ESR 28-joint disease activity score with erythrocyte sedimentation; REM remission; LDA low disease activity; MDA moderate disease activity; HDA high disease activity; IQR interquartile range

* DAS28-ESR at the 3-month visit was available in 51 patients from C1 cohort (82.3%), in 554 patients from C2 (92.6%), 113 patients from C3 (91.1%) and 476 patients from C4 (96.9%).
