# Supplementary material for: Switching first-line targeted therapy after not reaching low disease activity within 6 months is superior to conservative approach: a propensity score-matched analysis from the ATTRA registry
Source: Arthritis Res Ther. 2021 Jan 6;23:11. doi: 10.1186/s13075-020-02393-8 (PMC7789592; doi:10.1186/s13075-020-02393-8)
Supplement: Supplementary file 7 — Additional file 7: Supplementary Figure 3. Propensity score densities of cohorts C3 and C4 before and after matching. [file 13075_2020_2393_MOESM7_ESM.docx]

**Supplementary Figure 3** Propensity score densities of cohorts C3 and C4 before and after matching
